# Cross-modal correspondence between auditory pitch and visual elevation modulates audiovisual temporal recalibration

**DOI:** 10.1038/s41598-022-25614-3

**Published:** 2022-12-09

**Authors:** Kyuto Uno, Kazuhiko Yokosawa

**Affiliations:** 1 Department of Psychology, Graduate School of Humanities and Sociology, The University of Tokyo, 7-3-1 Hongo, Bunkyo-ku, Tokyo, 113-0033 Japan; 2 Japan Society for the Promotion of Science, 5-3-1 Kojimachi, Chiyoda-ku, Tokyo, 102-0083 Japan

**Keywords:** Human behaviour, Psychology

## Abstract

Cross-modal correspondences refer to associations between feature dimensions of stimuli across sensory modalities. Research has indicated that correspondence between audiovisual stimuli influences whether these stimuli are integrated or segregated. On the other hand, the audiovisual integration process plastically changes to compensate for continuously observed spatiotemporal conflicts between sensory modalities. If and how cross-modal correspondence modulates the “recalibration” of integration is unclear. We investigated whether cross-modal correspondence between auditory pitch and visual elevation affected audiovisual temporal recalibration. Participants judged the simultaneity of a pair of audiovisual stimuli after an adaptation phase in which alternating auditory and visual stimuli equally spaced in time were presented. In the adaptation phase, auditory pitch and visual elevation were manipulated to fix the order within each pairing of audiovisual stimuli congruent with pitch-elevation correspondence (visual leading or auditory leading). We found a shift in the point of subjective simultaneity (PSS) between congruent audiovisual stimuli as a function of the adaptation conditions (Experiment 1, 2), but this shift in the PSS was not observed within incongruent pairs (Experiment 2). These results indicate that asynchronies between audiovisual signals congruent with cross-modal correspondence are selectively recalibrated.

## Introduction

The human brain combines signals from multiple sensory modalities and appropriately develops coherent perceptions and cognitions^1^. In this process, the spatiotemporal proximity and correlation between signals and relationships between different feature dimensions are used as informative integration cues^2,3^. Associating feature dimensions of stimuli across sensory modalities is called cross-modal correspondence^3^. For example, when presented with two meaningless figures, one rounded and the other pointed, and asked to identify which is “Bouba” and which is “Kiki,” the majority of people associate the rounded figure with “Bouba” and the pointed figure with “Kiki” (Bouba/Kiki effect^4^). Cross-modal correspondence has also been reported between simpler feature dimensions. Notably, many studies have reported associations between the pitch of auditory stimuli and several characteristics of visual stimuli such as the vertical position, brightness, and size^5^.

The mechanisms by which cross-modal correspondence affects low-level multisensory perception have long been debated. Recent studies have shown that several types of cross-modal correspondences influence audiovisual intersensory binding^6–9^, although other studies have found no such effects^10,11^. For example, Parise and Spence^9^ reported that the spatiotemporal discrimination sensitivity of visual and auditory stimuli decreased when they were congruent (e.g., higher pitch tones—smaller circles) compared to when they were incongruent with cross-modal correspondence (higher pitch tones—larger circles). This finding indicates that the spatiotemporal binding window is wider for stimulus pairs congruent with cross-modal correspondence and suggests that the human perceptual system automatically uses cross-modal correspondence as a cue for inferring audiovisual signals’ source when deciding whether multiple sensory signals should be integrated or segregated.

The above-discussed studies have focused on cross-modal correspondence effects on immediate audiovisual interactions. However, audiovisual intersensory binding does not occur similarly at all time points. Because prolonged exposures to spatiotemporal conflicts between audiovisual signals induce compensatory aftereffects, not only the immediate effects but also aftereffects must be considered to fully understand audiovisual intersensory binding mechanisms^12^. However, the role of cross-modal correspondence in aftereffects has remained unclear.

We focused on adapting to the time lag between audiovisual signals to examine the role of cross-modal correspondence in aftereffects. The adaptation process to such time lags, which is called “temporal recalibration,” refers to the phenomenon in which prolonged exposure to asynchronies between auditory and visual signals modulates the point of subjective simultaneity (PSS) between subsequent audiovisual signals such that asynchronous signals seem to be more synchronous^13,14^. Recent studies have suggested that temporal recalibration is modulated by spatial grouping between audiovisual adaptors^15^ and selective attention^16^ when multiple audiovisual events compete for recalibration. The participants in the study by Yarrow et al.^15^ were exposed to a train of alternating flashes and tones, equally spaced in time on the left and right sides of the fixation. The order of flashes and beeps on either side of the fixation was constant under each of the two conditions. The light lagging condition consisted of multiple repetitions of the following sequence: left tone–left light–right tone–right light. Moreover, the light leading condition consisted of multiple repetitions of the following sequence: left tone–right light–right tone–left light. They reported that the PSS between subsequent lights and tones differed between the two conditions to compensate for the time lag between stimuli at the same location, suggesting that spatial cues modulate temporal recalibration when audiovisual stimuli are not grouped according to temporal proximity. Ikumi and Soto-Faraco^16^ tested temporal recalibration following an adaptation phase. They presented two opposing audiovisual asynchronies (i.e., flash–sound–flash) and reported that the PSS differed based on whether observers focused their visual attention on the first or the second flash, suggesting that selective attention modulates the direction of temporal recalibration. These findings raise the possibility that spatial proximity^15^ and/or selective attention^16^ assist the perceptual system in comprehending causal structures in a multitude of sensory inputs having temporal proximity when recalibrating temporal discrepancy between audio and visual signals assumed to originate from the same unitary event. Based on this idea, we hypothesized that audiovisual stimuli congruent with cross-modal correspondence are grouped among multiple signals that have temporal proximity to each other, and temporal recalibration occurs according to the stimulus order within groups.

We investigated the effect of pitch-elevation correspondence, the tendency to associate higher/lower pitch tones with upper/lower visual positions, on temporal recalibration to test this hypothesis. Previous studies, using different types of psychophysical tasks such as speeded target detection^17,18^, speeded classification^19–24^, and temporal order judgment^7,11^, have sometimes shown that pitch-elevation correspondence impacts human perceptual processing. In addition, recent studies^25,26^ indicated that higher pitch tones are more likely to be emitted from upper positions and that pitch-elevation correspondence develops from statistical learning of causal structures in natural auditory scenes. For these reasons, we hypothesized that pitch-elevation correspondence might also affect audiovisual temporal recalibration.

**Figure 1:**
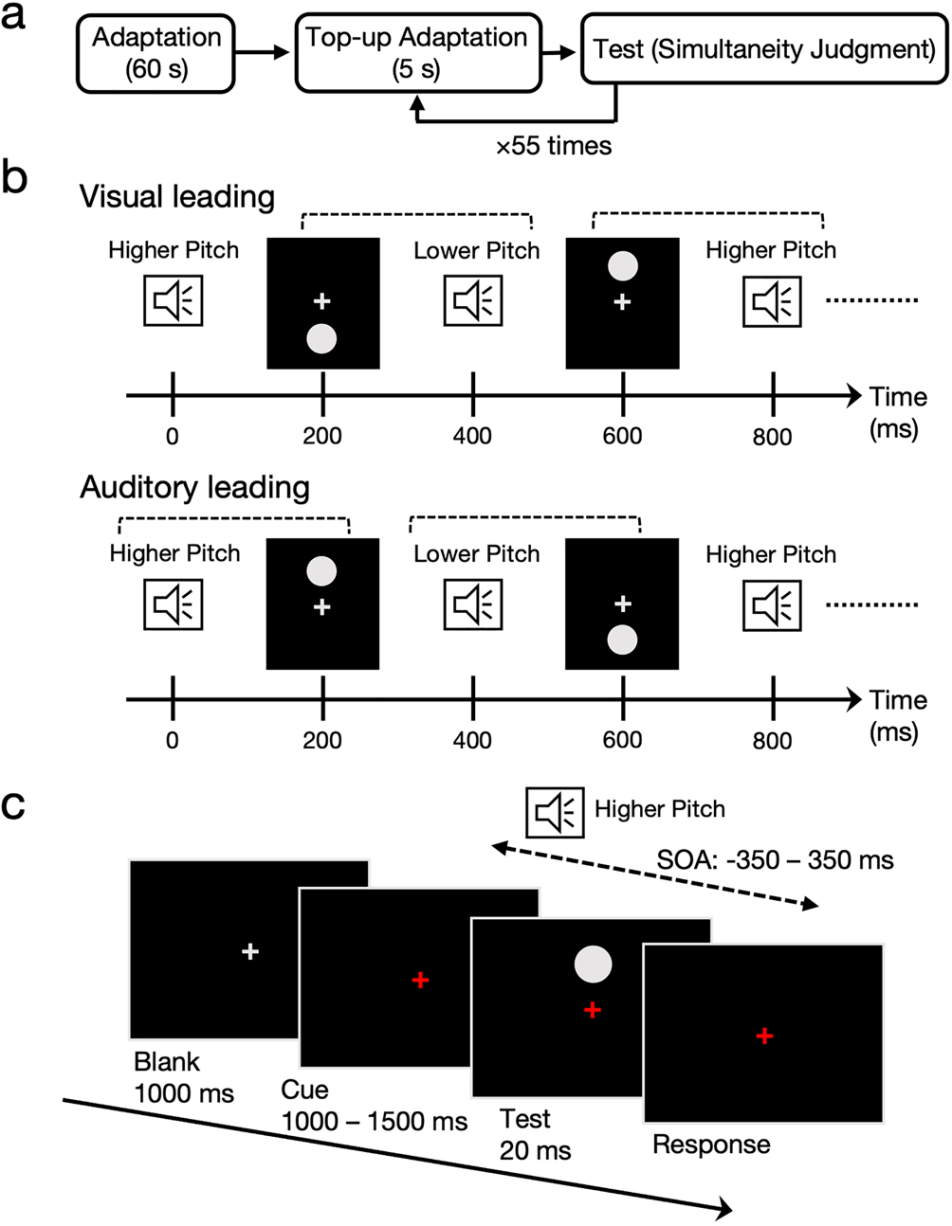
Design and procedure of Experiment 1. (**a**) Timeline of the experimental procedure in each block. (**b**) Schematic illustration of the two adaptation conditions. Audio and visual stimuli were presented alternatively with grouping implied by the congruency with cross-modal correspondence between auditory pitch and visual elevation (shown as dotted lines). (**c**) The procedure of a single test trial (a simultaneity judgment task).

We developed the adaptor sequence used in this study based on Yarrow et al.^15^ (see Fig. 1b). Participants were exposed to a train of alternating visual (circles) and auditory stimuli (tones) equally spaced in time. We regarded audiovisual stimuli in the adaptation sequence congruent with pitch-elevation correspondence as pairs and manipulated the order within pairs of audiovisual stimuli. We always presented a visual stimulus before an auditory stimulus within each congruent pairing in the visual leading condition. On the other hand, we always presented an auditory stimulus before a visual stimulus within each congruent pairing in the auditory leading condition. In Experiment 1, we examined whether observers adapted to the order of audiovisual stimuli congruent with pitch-elevation correspondence and showed shifts in the perceived simultaneity in the subsequent simultaneous judgments for congruent audiovisual pairs. In Experiment 2, we examined whether we could replicate the results in Experiment 1 and whether the identical adaptation also caused shifts in the perceived simultaneity of pitch-elevation correspondence incongruent audiovisual pairs.

## Experiment 1

### Methods

#### Participants

We recruited 20 participants (10 women, 10 men; mean age: 22.0 years, *SD* = 1.6) who reported normal or corrected-to-normal vision and hearing. We predetermined the sample size (*N* = 20) such that it was at least as large as previous studies’ sample sizes (*N* = 12 in Yarrow et al.^15^; *N* = 14 and 19 in Experiment 1 and 2 in Ikumi & Soto-Faraco^16^, respectively). We did not conduct a power analysis to determine this experiment’s sample size. However, the sample size we decided on allowed us to detect an effect size of *d*_*z*_ (the mean difference between conditions divided by its unbiased standard deviation) = 0.66 with α = 0.05 (two-tailed) and power = 80% (calculated by G*Power Version 3.1^27^), which is much smaller than the effect size estimated from the published *t*-value in Yarrow et al.^15^ (*d*_*z*_ = 1.30).

All participants were naïve about the study. They were paid 2,040 Japanese yen (approximately $20) to participate for two hours. The study was conducted according to the guidelines of the Declaration of Helsinki. It was approved by the Ethical Committee of the Department of Psychology at the University of Tokyo. Informed consent was obtained from all the participants before taking part in the study.

#### Apparatus

We ran the experiment on a PC using the Psychophysics Toolbox^28–30^ in MATLAB (The MathWorks, Inc). Visual stimuli were presented on a calibrated colour monitor (Mitsubishi Diamondtron VX920) having a resolution of 1024 × 768 pixels and a refresh rate of 100 Hz. Participants viewed the monitor binocularly from a distance of 57 cm, with their heads resting on a chinrest in a quiet, dark room. Auditory stimuli were presented monaurally through headphones (Sennheiser HDA300). Accurate stimulus timing was confirmed using a digital oscilloscope. Participants responded using a numeric keyboard.

#### Stimuli

The visual stimuli consisted of light grey circles subtending 3.0° of visual angle, presented 3.0° above or below a light grey central fixation point (0.5° in width and height) against a black background. Individual visual stimuli were presented for 20 ms. The auditory stimuli consisted of 20 ms pure tones, with 4 ms linear ramps at onset and offset. The lower pitch tone had a 1000 Hz frequency, and the higher pitch tone had a 4000 Hz frequency. We adjusted the higher and lower pitch tones to be equally loud (approximately 70 dB measured inside the headphone ear cup).

#### Design and procedures

A block of trials is depicted in Fig. 1a. Each block consisted of an initial adaptation phase, top-up adaptation phases, and test phases. Each participant completed eight blocks of trials. We instructed the participants to maintain their fixation on the central fixation cross throughout the experiment.

Each block began with a 60 s initial adaptation phase, in which we repeatedly presented two visual and two auditory stimuli patterns. As shown in Fig. 1b, there were two adaptation conditions (visual leading and auditory leading), similar to the experimental design in Yarrow et al.^15^. The adaptation train in the visual leading condition contained multiple repetitions of the following sequence: higher pitch tone-lower located circle–lower pitch tone–upper located circle, such that a visual stimulus was always presented before an auditory stimulus within each pairing of audiovisual stimuli congruent with pitch-elevation correspondence. The adaptation train in the auditory leading condition contained multiple repetitions of the following sequence: higher pitch tone–upper located circle–lower pitch tone–lower located circle, such that an auditory stimulus was always presented before a visual stimulus within each congruent pairing. The stimulus onset asynchronies (SOAs) between audio and visual stimuli were physically identical (200 ms) in both conditions to remove temporal cues for bisensory events’ grouping. The duration of each audiovisual stimulus was constant at 20 ms.

After the initial adaptation phase, participants repeated the combination of the top-up adaptation phase and the test phase 55 times (Fig. 1a). The top-up adaptation sequence, which we presented for 5 s (i.e., 6.25 repetitions of the four-stimulus patterns), was identical to that in the initial adaptation phase. After that, the colour of the central fixation cross changed from light grey to red. Then, the participants engaged in the test phase (a simultaneity judgment (SJ) task, see Fig. 1c). We presented the test audio and visual stimuli, and we requested the participants to judge whether these stimuli were synchronous (i.e., simultaneous) or asynchronous. After the participants responded, we again presented the top-up adaptation sequence.

In the test phase, the participants responded by pressing one of two computer keys (“4” or “6” on the numeric keypad). We counterbalanced the key assignments across participants. The stimulus onset asynchronies (SOAs) between the two test stimuli were manipulated across 11 levels (± 350, ± 250, ± 150, ± 100, ± 50, and 0 ms, with negative numbers indicating an auditory stimulus presented before a visual stimulus). The pairing of audio and visual stimuli in the test phase was always congruent with pitch-elevation correspondence (i.e., higher pitch tone–upper located circle or lower pitch tone–lower located circle).

Each participant completed four blocks of trials under each adaptation condition. We alternated the two adaptation conditions in every two blocks and counterbalanced the starting condition across participants. A higher pitch tone and an upper located circle were used as audiovisual stimuli in the test phase of the four blocks, whereas we used a lower pitch tone and a lower located circle in the other four blocks. The participants took a break for a few minutes after each block. Each participant responded to 440 test trials (55 trials × 8 blocks): 11 SOAs (randomized order across trials) × two types of pairings of stimuli in the test phase × two adaptation conditions × 10 occasions.

#### Data analysis

We calculated the proportion of simultaneous responses for each SOA condition under the two adaptation conditions for each participant. Then, the data were fitted by maximum-likelihood estimation with a model constructed from the differences in two cumulative Gaussians using MATLAB (MathWorks, Inc) such that it was appropriate for fitting data from simultaneity judgment (SJ) tasks^31–34^

1 $$\begin{array}{c}p\left(\mathrm{simultaneous}\right)= \Phi \left({\mathrm{C}}_{\mathrm{High}},\mathrm{ SOA}, {\upsigma }_{\mathrm{High}}\right)- \Phi \left({\mathrm{C}}_{\mathrm{Low}},\mathrm{ SOA}, {\upsigma }_{\mathrm{Low}}\right)\end{array}$$

In which Φ is the normal cumulative distribution function. The C_High_ and C_Low_ parameters represent the mean positions of the decision criteria on the SOA, and the σ_High_ and σ_Low_ parameters represent the slope on each side of the function. The point of subjective simultaneity (PSS) in each condition for each participant was then calculated as the average of the simultaneity criteria for audio-leads and visual-leads SOA^31^:

2 $$\begin{array}{c}PSS= \frac{{\mathrm{C}}_{\mathrm{High}}+{\mathrm{C}}_{\mathrm{Low}}}{2}\end{array}$$

Positive/negative PSSs indicate that audiovisual stimuli are more likely to be judged as simultaneous when a visual stimulus is presented before/after an auditory stimulus.

Our primary interest was in differences in PSS across conditions. We also examined whether the range of SOAs judged to be simultaneous varied across conditions. We calculated the window of subjective simultaneity in each condition for each participant as the difference between the two simultaneity criteria for audio-leads and visual-leads SOA.

Participants who could not adequately discriminate the simultaneity of audiovisual stimuli in this experiment needed to be excluded from the analysis. Therefore, we assessed whether the four-parameter model fitted better than a simpler two-parameter model (a single cumulative Gaussian)^31^ for each participant in each condition using the following formula.

3 $$\begin{array}{c}p\left(\mathrm{simultaneous}\right)= \Phi \left(\mathrm{C},\mathrm{ SOA},\upsigma \right)\end{array}$$

We estimated the deviance for each model fit and retained participants only when the difference in deviance from a two-parameter model to a four-parameter model in each of the two conditions was significantly greater than the critical values for the chi-square distribution with 2 degrees of freedom^31,34^. Based on this criterion, none of the participants were excluded. Therefore, all 20 participants in Experiment 1 were included in the subsequent analysis.

The above-described fitting method has been proposed recently, and it was common to fit a normal distribution function to the data from audiovisual simultaneity judgment tasks^35^ until the introduction of the above method. Therefore, we conducted our analysis on PSS not only by the above method but also by fitting a normal distribution function and confirmed that the main conclusion of this experiment did not change (see Supplementary information for details).

### Results and discussion

**Figure 2:**
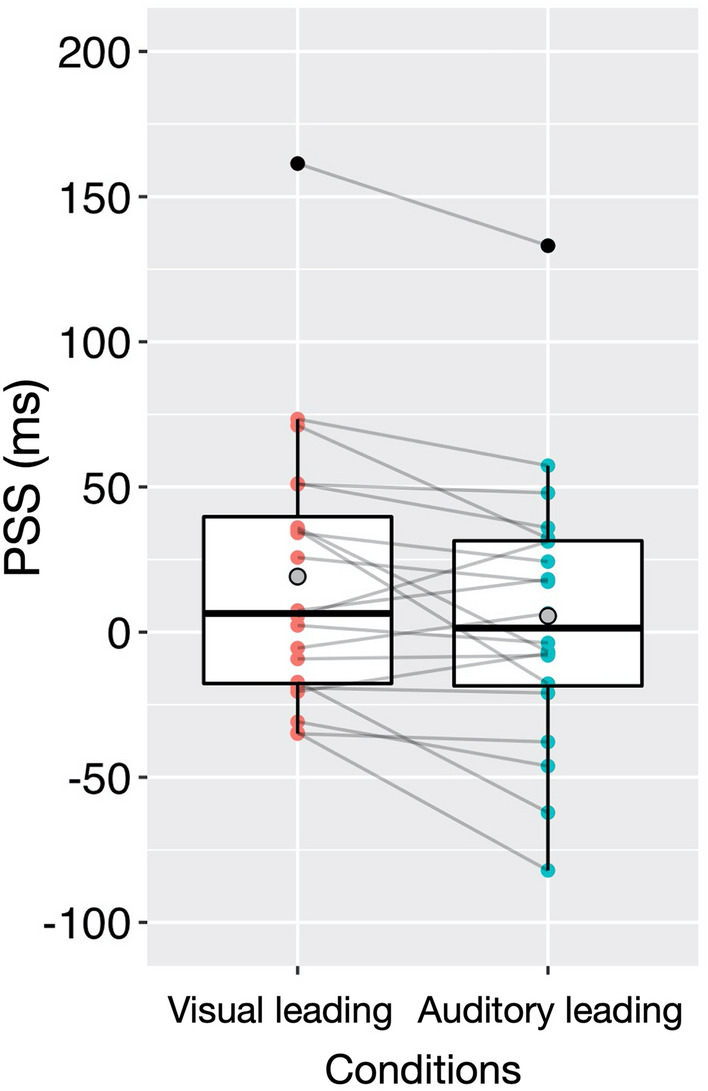
PSS data as a function of the adaptation condition (visual leading or auditory leading) in Experiment 1. Boxes represent the interquartile ranges (IQRs), central horizontal lines the medians, and grey circles the means. Magenta and cyan points represent the PSS data of individual participants. The vertical lines represent the ranges from “the first quartile − 1.5 × IQR” to “the third quartile + 1.5 × IQR,” and black points indicate the PSS data outside the ranges (i.e., outliers).

Figure 2 presents participants’ PSSs under the visual leading vs. auditory leading conditions of Experiment 1. We used a standard two-tailed parametric test to assess differences in PSSs across the two adaptation conditions. The results indicated that there was a significant difference (13.5 ms on average) between the PSS in the visual leading (19.1 ms on average) and the auditory leading conditions (5.6 ms on average; *t*(19) = 2.69, *p* = 0.014, *d*_*z*_ = 0.60), indicating pitch-elevation correspondence in adaptation sequences influenced subsequent audiovisual simultaneity judgments. The difference between the conditions was significant even when the outliers shown in Fig. 2 were excluded (see Supplementary information for details). We also conducted a *t*-test to assess differences in the windows of subjective simultaneity across the two conditions. However, the *t*-test indicated that the difference between the window in the visual leading (446.7 ms on average) and the auditory leading conditions (456.5 ms on average) was not statistically significant (*t*(19) = 0.63, *p* = 0.539, *d*_*z*_ = 0.14).

These results supported the hypothesis that audiovisual stimuli congruent with pitch-elevation correspondence are grouped among multiple signals that are temporally close to each other and that temporal recalibration occurs according to the stimulus order within groups. This experiment did not clarify whether temporal recalibration occurred under both (auditory leading and visual leading conditions) or only one adaptation condition because we did not take a baseline measure. However, either possibility would lead to the conclusion that pitch-elevation correspondence congruency modulates temporal recalibration (see also “General discussion”).

In Experiment 1, we tested the perceived simultaneity of audiovisual pairs congruent with pitch-elevation correspondence after adaptation. Experiment 2 investigated whether the PSS shifts following adaptation observed in Experiment 1 were specific to audiovisual pairs congruent with pitch-elevation correspondence or generalized to other pairs. We examined the effects of the identical adaptation sequences as in Experiment 1 on the perceived simultaneity of audiovisual pairs incongruent with pitch-elevation correspondence.

## Experiment 2

The purpose of Experiment 2 was to determine whether shifts in audiovisual pairs’ PSSs following adaptation occur only in audiovisual stimuli pairs congruent with pitch-elevation correspondence, or other types of stimuli as well. We expected no difference in the PSS after adaptation in incongruent pairs if temporal recalibration according to pitch-elevation correspondence-based grouping occurred selectively for congruent stimulus pairs. On the other hand, if the recalibration generalized to other types of audiovisual pairs, we could expect a similar PSS shift for incongruent and congruent pairs.

### Methods

#### Participants

Thirty-two participants reporting normal or corrected-to-normal vision and hearing that did not participate in Experiment 1 took part in Experiment 2 (17 women and 15 men; mean age: 21.0 years, *SD* = 5.0). The effect size of the adaptation condition's main effect on PSSs in Experiment 1 was *d*_*z*_ = 0.60 when we included outliers and *d*_*z*_ = 0.56 when we excluded outliers. The required sample size to detect an effect of *d*_*z*_ = 0.56 with *α* = 0.05 (two-tailed) with a power of 80% would be 28 (calculated by G*Power Version 3.1^27^). The desired number of participants for counterbalancing stimulus conditions was a multiple of 8. Therefore, we decided to include 32 participants in Experiment 2.

All participants were naïve about the study and were paid 4,080 Japanese yen (approximately $40) to participate for four hours. The other requirements of this study were identical to those of Experiment 1.

#### Apparatus and stimuli

The apparatus and stimuli used in Experiment 2 were identical to Experiment 1.

#### Design and procedures

In Experiment 2, we manipulated the cross-modal congruency of audiovisual stimuli in the test phase (simultaneity judgment tasks) and the type of adaptation conditions (visual leading vs. auditory leading). The pairing of audio and visual stimuli in the test phase was either congruent (i.e., higher pitch tone–upper located circle or lower pitch tone–lower located circle) or incongruent (i.e., higher pitch tone–lower located circle or lower pitch tone–higher located circle) with pitch-elevation correspondence. Therefore, 880 test trials (55 trials × 16 blocks), twice as many as in Experiment 1, were required for each participant: 11 SOAs × four types of pairings of stimuli in the test phase × two adaptation conditions × 10 occasions. The order of 11 SOAs and four types of stimuli pairings in the test phase were independently randomized across trials. Each participant engaged in the experiment for two days and completed 8 blocks (440 trials) per day. We alternated the two adaptation conditions in every four blocks. Moreover, we independently counterbalanced the starting condition on each day across participants so that there were four different adaptation condition orders: VA-VA, VA-AV, AV-VA, AV-AV (V represents four blocks of visual leading condition, and A represents four blocks of auditory leading condition).

**Figure 3:**
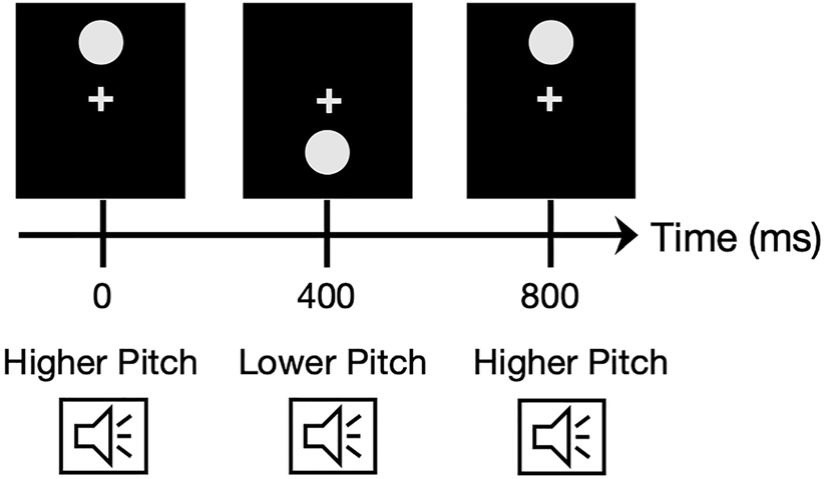
Schematic illustration of the stimulus observation phase of Experiment 2.

We also made two modifications to Experiment 1 described below. First, the SOAs between audiovisual stimuli in the test phase of Experiment 1 had 11 levels: ± 350, ± 250, ± 150, ± 100, ± 50, and 0 ms, which were modified in Experiment 2: ± 400, ± 320, ± 240, ± 160, ± 80, and 0 ms. We slightly widened the SOAs range because several participants reported that nearly all audiovisual stimuli were seemingly simultaneous in Experiment 1. Second, we included a stimulus observation phase to strengthen the pitch-elevation correspondence before the start of each block (Fig. 3). The participants were exposed to 440 incongruent stimulus pairs throughout the experiment because we presented audiovisual pairs incongruent with pitch-elevation correspondence during the test phase. We considered the possibility that repeated exposure of incongruent pairs might weaken existing cross-modal correspondences between auditory pitch and visual elevation because cross-modal mappings between two sensory signals can vary by learning their statistical co-occurrence^36^. We then attempted to eliminate this possibility by presenting a series of congruent stimulus pairs. Two types of audiovisual pairs congruent with pitch-elevation correspondence (i.e., higher pitch tone–upper located circle and lower pitch tone–lower located circle) were presented alternately every 400 ms in the stimulus observation phase. The audiovisual stimuli within each pair were synchronized, and participants were asked to observe them for 60 s before starting each block.

#### Data analysis

We estimated the PSS and the window of subjective simultaneity under each condition for each participant by fitting the four-parameter model that was identical to Experiment 1. We excluded one participant from the subsequent analysis based on the identical exclusion criterion as in Experiment 1. We also analysed the PSS data of Experiment 2 by fitting the normal distribution function similar to Experiment 1, which confirmed that the main conclusion of Experiment 2 did not change (see Supplementary information for details).

### Results and discussion

**Figure 4:**
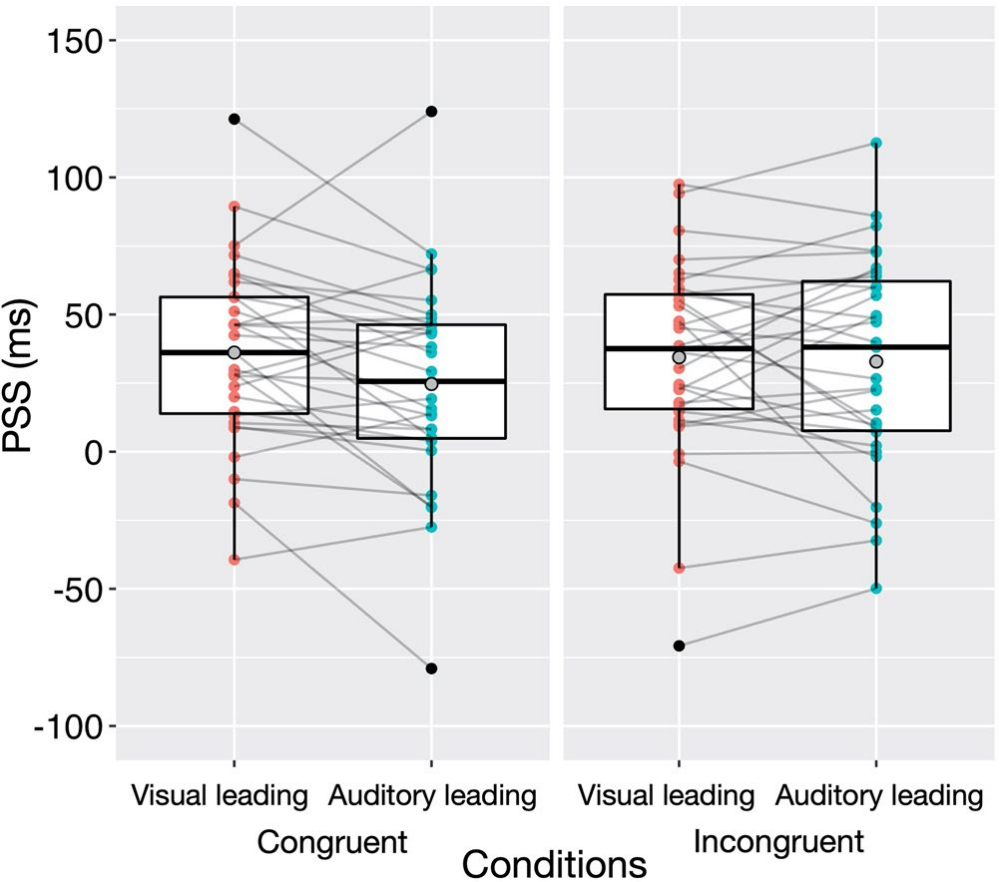
PSS data as a function of test stimulus condition (congruent or incongruent) and adaptation condition (visual leading or auditory leading) in Experiment 2.

The PSS values were subjected to a repeated-measures analysis of variance (ANOVA), with test stimulus condition (congruent and incongruent) and adaptation condition (visual leading and auditory leading) as within-subject variables (see Fig. 4). The results indicated that neither the main effect of test stimulus condition (*F*(1, 30) = 1.21, *p* = 0.279, ${\eta }_{p}^{2}$ = 0.039), nor the main effect of adaptation condition were significant (*F*(1, 30) = 2.99, *p* = 0.094, ${\eta }_{p}^{2}$ = 0.091). However, the interaction between these conditions was significant (*F*(1, 30) = 5.00, *p* = 0.033, ${\eta }_{p}^{2}$ = 0.143). A post-hoc simple effect test of the interaction revealed a significant simple main effect of the adaptation condition in the congruent test stimulus condition (*F*(1, 30) = 6.58, *p* = 0.016, ${\eta }_{p}^{2}$ = 0.180). Consistent with Experiment 1, there was a significant difference (11.4 ms on average) between the PSS in the visual adaptation condition (36.1 ms on average) and the auditory adaptation condition (24.7 ms on average) when audiovisual stimuli in the test phase were congruent with pitch-elevation correspondence. On the other hand, the simple main effect of adaptation condition in the incongruent test stimulus condition was not significant (*F*(1, 30) = 0.13, *p* = 0.716, ${\eta }_{p}^{2}$ = 0.005). Moreover, the simple main effect of the test stimulus condition was significant in the auditory leading condition (*F*(1, 30) = 4.79, *p* = 0.037, ${\eta }_{p}^{2}$ = 0.138) but not in the visual leading condition (*F*(1, 30) = 0.29, *p* = 0.596, ${\eta }_{p}^{2}$ = 0.011). The results pattern in Experiment 2 was nearly identical when the outliers shown in Fig. 4 were excluded (see Supplementary information for details).

We also conducted a two-way ANOVA for the windows of subjective simultaneity. However, the ANOVA indicated that neither the main effects nor the interaction were significant (the main effect of test stimulus condition: *F*(1, 30) = 2.57, *p* = 0.120, ${\eta }_{p}^{2}$ = 0.079; the main effect of adaptation condition: *F*(1, 30) = 0.49, *p* = 0.490, ${\eta }_{p}^{2}$ = 0.016; the interaction between these conditions: *F*(1, 30) = 0.45, *p* = 0.508, ${\eta }_{p}^{2}$ = 0.015).

Experiment 2 replicated the finding of Experiment 1 that pitch-elevation correspondence in adaptation sequences influences the PSS of audiovisual pairs when the test stimulus pairs are congruent with pitch-elevation correspondence. Experiment 2 further indicated that this effect could be observed only for congruent test stimulus pairs. The PSS of incongruent audiovisual stimulus pairs did not differ significantly between the two adaptation conditions, indicating the possibility that the adaptation sequences did not modulate the PSS of incongruent audiovisual stimulus pairs. We have discussed this result in detail in the “General discussion”.

Unlike in Experiment 1, in Experiment 2 we included a stimulus observation phase to strengthen the pitch-elevation correspondence before the start of each block. This manipulation might have an impact on the simple main effect of the adaptation condition in the congruent test stimulus condition. The size of this effect (${\eta }_{p}^{2}$ = 0.180, that is, *d*_*z*_ = 0.47) was similar to or slightly smaller than the effect size of the adaptation condition on the PSS in Experiment 1 (*d*_*z*_ = 0.60). Based on this, it would be possible that repeated exposure of congruent audiovisual pairs during the stimulus observation phase offset the effect of repeated exposure of incongruent pairs during the test phase, resulting in the shift of PSS. On the other hand, repeated exposure of congruent pairs in the stimulus observation phase did neither increase the amount of recalibration compared to Experiment 1 nor change the window of subjective simultaneity between congruent and incongruent pairs in the test phase. This suggests that the stimulus observation phase had, if any, a limited effect on the results in Experiment 2.

In Experiment 2, one participant was excluded and was not replaced to maintain counterbalancing. Therefore, we cannot rule out the possibility that this might have affected the results. However, the deviation from complete counterbalancing was so slight that any practice and fatigue effects on PSS would have to be very large to affect the results significantly. However, it is hard to assume that these effects would be huge. Therefore, we considered that incomplete counterbalancing did not impact the main results of this study significantly.

## General discussion

We investigated the effects of cross-modal correspondence between auditory pitch and visual elevation on temporal recalibration. We presented participants with two types of adaptation sequences in which visual and auditory stimuli were presented alternately. The intervals between stimuli were always equal, making it unlikely that specific audiovisual stimuli would be grouped according to temporal proximity. We manipulated the vertical position of the visual stimuli and the pitch height of the auditory stimuli in each adaptation sequence. We assumed that audiovisual stimuli consistent with the pitch-elevation correspondence would be grouped together. Within each group, the visual stimulus always preceded the auditory stimulus in the visual leading condition’s sequence, and the auditory stimulus always preceded the visual stimulus in the auditory leading condition’s sequence. We measured participants’ audiovisual simultaneity judgments after each adaptation sequence and demonstrated the following. Firstly, the points of subjective simultaneity (PSSs) to pairs of audiovisual stimuli consistent with pitch-elevation correspondence varied according to the adaptation sequence in Experiments 1 and 2. This finding indicated that the PSS was larger after observing the visual leading condition’s sequence than after observing the auditory leading condition’s sequence. Therefore, we suggest that temporal recalibration might have occurred according to the stimulus order of the audiovisual groups, which was consistent with pitch-elevation correspondence.

On the other hand, Experiment 2 indicated that the effect of the adaptation sequence depended on the congruency of test stimulus pairs to pitch-elevation correspondence. This is because no significant difference was observed in the mean PSSs between adaptation sequence conditions for audiovisual stimuli pairs incongruent with pitch-elevation correspondence. These results suggest that pairs of audiovisual stimuli consistent with pitch-elevation correspondence might be grouped together from multiple signals temporally close to each other and that time lags between these stimuli might be selectively compensated according to the order of presentation within the group.

As mentioned in the “Results and discussion” section of Experiment 1, the results of this study showing that the PSSs for audiovisual stimulus pairs consistent with the pitch-elevation correspondence differed between two types of adaptation sequences does not necessarily mean that selective recalibration based on pitch-elevation correspondence occurred in both two adaptation conditions. We did not make baseline measurements in either Experiment 1 or 2. Therefore, it is impossible to estimate shifts in PSSs from pre- to post-adaptation under each condition from these experiments’ results. However, the results indicated that temporal recalibration depends on the audiovisual stimulus grouping based on pitch-elevation correspondence at least under these experimental situations, even though it was impossible to determine whether such adaptation occurred under each condition. The only difference between the two adaptation conditions was the audiovisual stimuli’s order (i.e., the consistency of successive audiovisual stimuli for pitch-elevation correspondence). Therefore, the significant difference in the PSS after adaptation between the two conditions indicates that the grouping effect based on pitch-elevation correspondence modulated observers’ simultaneity judgments. Moreover, the mean PSS in the visual leading condition was larger than that in the auditory leading condition, indicating that this modulation occurred in a direction that compensated for the inter-stimulus interval within the groups. Again, such compensation for the time lag (i.e., temporal recalibration relative to pre-adaptation) could have occurred either in both or just one of the adaptation conditions. Future studies comparing pre- with post-adaptation are needed to comprehensively understand the mechanisms of recalibration based on cross-modal correspondence demonstrated in this study.

### Possible descriptive mechanisms

Three major descriptive models have been proposed for the mechanism of audiovisual temporal recalibration^37–39^. The first is the “latency-shift model,” which postulates that adaptation changes perceptual latency for individual modalities^40,41^. In this model, the asynchrony between modalities is compensated by changing the sensory latency in the relatively less reliable modality to match the sensory latency in the more reliable modality^40^. The second model is the “population-coding model,” which assumes a population of neurons that respond selectively to audiovisual asynchrony of a particular range^42^. In this case, repeated exposure to a constant time lag between audiovisual stimuli is thought to alter the perception of relative timing by reducing the response gain of neurons that are selective for that time lag. The third model is the “criterion-change model,” suggesting that repeated exposure to asynchrony does not change the sensory processing of timing but changes the observer’s simultaneity judgment criteria instead^32^. A recent study^38^ has shown that temporal recalibration caused by prolonged exposure to isolated pairs of audiovisual stimuli (i.e., the interval between each presentation of an audiovisual pair greatly exceeds the interval between the audiovisual stimuli within each pair) cannot be explained by changes in decision criteria only but would be explained by combining the “latency-shift model” and the “population-coding model.”

The present study indicated that the effect of the adaptation sequence depends on the congruency of test stimuli to pitch-elevation correspondence. However, it remains unclear whether such selective recalibration based on relationships between stimulus properties shown in the present study occurs through the identical mechanism as recalibration due to repeated exposure to isolated pairs of audiovisual stimuli. In this study, the relationship between test stimuli’s properties determined whether the time lag between a pair of audiovisual stimuli was compensated, suggesting the primary involvement of top-down factors in the temporal recalibration process. Further investigations of these mechanisms are needed to elucidate the temporal recalibration processes fully.

### Functional significance

The compensation for the time lag between audiovisual signals might contribute to integrating these signals and perceiving them as simultaneously originating from a single object or event. In other words, if the two signals originate from the same source, then temporal recalibration would facilitate accurate perception of the external world. However, temporal recalibration would increase the risk of causing false multisensory integration if the two signals originated from two different events. Therefore, it is possible that given multiple audiovisual signals, temporal recalibration could be selectively modulated according to the observer’s prior knowledge of the signal sources if the recalibration process were adaptive to the external environment.

The findings of the present study are consistent with this hypothesis. Previous studies have shown that pitch-elevation correspondence reflects statistical properties (co-occurrence probability) of audiovisual signals in the external world^25,26^ and is considered to affect the “unity assumption” between audiovisual signals^2^. Therefore, pitch-elevation correspondence could provide a cue for grouping stimuli with a high probability of being from the same source among multiple signals close in time. Moreover, the point of subjective simultaneity shifted only for stimulus pairs congruent with the correspondence, supporting the view that time lags between signals presumed to originate from the same source are selectively recalibrated.

Other studies have shown that multiple temporal recalibrations can occur depending on the stimulus pair^43–47^. These studies used two types of audiovisual stimulus pairs defined by spatial location and/or stimulus features (e.g., male voice and video during speech, and female voice and video during speech). In one stimulus pair, the auditory stimulus always preceded the visual stimulus, and in the other, the visual stimulus always preceded the auditory stimulus. When these stimulus pairs were temporally isolated and repeatedly presented, temporal recalibration was found to occur for each pair suggesting that temporal recalibration can occur selectively, which is consistent with the present study’s findings. On the other hand, the stimulus pairs were presented in a temporally isolated manner so that the stimulus pairs were grouped based on temporal proximity in these studies. The present study demonstrated that pitch-elevation correspondence could be a cue for determining asynchronies between audiovisual stimuli that should be recalibrated when temporal proximity is not a grouping cue. These findings, taken together, suggest that temporal recalibration could play a functional role in constructing a coherent multisensory perception based on inferred causal structures in the complex external world where many signals are in spatiotemporal proximity.

A limitation of this study is that it did not directly manipulate the participants’ prior knowledge of the external world’s statistical properties. As mentioned earlier, this study suggests that pitch-elevation correspondence might be a cue for grouping audiovisual stimuli among multiple signals based on their causality. However, the pitch-elevation correspondence does not necessarily reflect only statistical properties. For example, pitch and elevation can be assigned the standard linguistic labels, “high” and “low,” indicating the possibility that cross-modal correspondence is also semantically mediated^3^. It is necessary for future studies to experimentally manipulate the co-occurrence probability of audiovisual stimuli and examine the effect on temporal recalibration and clarify whether and how selective temporal recalibration reflects the observer’s prior knowledge of the external world.

### Conclusion

This study’s results indicate that cross-modal correspondence between auditory pitch and visual elevation can be used as a cue for promoting audiovisual pairings and selectively recalibrate asynchronies between grouped audiovisual stimuli. Previous studies have indicated that cross-modal correspondences can be used as a cue for deciding whether transient audiovisual signals should be integrated or segregated. The findings of the current study provide new evidence that cross-modal correspondence can also play a role in the process of experience-based changes in audiovisual integration. This finding supports the idea that temporal recalibration is functionally significant because it facilitates the integration of multisensory signals originating from the same source.

## Supplementary Information

Supplementary Information.

## Data Availability

The data and supplemental materials that support this study are available at https://osf.io/ngsk9/. The experimental programs and analysis codes are not available online but from the corresponding author on request.
