# Supplementary material for: Cross-modal correspondence between auditory pitch and visual elevation modulates audiovisual temporal recalibration
Source: Sci Rep. 2022 Dec 9;12:21308. doi: 10.1038/s41598-022-25614-3 (PMC9734665; doi:10.1038/s41598-022-25614-3)
Supplement: Supplementary file 1 — Supplementary Information. [file 41598_2022_25614_MOESM1_ESM.pdf]

- Supplementary Information -

Cross-modal correspondence between auditory pitch and visual elevation modulates audiovisual temporal recalibration

Kyuto Uno[1, 2, *] and Kazuhiko Yokosawa[1]

[1] Department of Psychology, The University of Tokyo, Japan

[2] Japan Society for the Promotion of Science, Japan

*kunopsy@gmail.com

Effects of outliers on the results of Experiment 1

We investigated the impact of outliers in the data by examining whether the results of Experiment 1 would change if we excluded outliers from the analysis. We defined an outlier in each condition to be any PSS data outside the following range:

$$[\mathrm{Q}_1 - 1.5(\mathrm{Q}_3 - \mathrm{Q}_1), \mathrm{Q}_3 + 1.5(\mathrm{Q}_3 - \mathrm{Q}_1)] \qquad \text{(S1)}$$

where Q_1 is the first quartile and Q_3 is the third quartile. One participant's data in visual leading and auditory leading conditions were considered outliers and excluded from the subsequent analysis of the data based on this criterion (see also Figure 2 in the main text).

We used a standard two-tailed parametric test to assess differences in PSSs across the two adaptation conditions. There was a significant difference between the PSS in the visual leading condition and the auditory leading condition ($t(18) = 2.44$, $p = .025$, $d_D = 0.56$), similar to when we did not exclude the outliers. Therefore, we concluded that the presence of outliers would not affect the overall conclusions of Experiment 1.

Effect of changing the fitting method on the results of Experiment 1

To examine the effect of different fitting methods on Experiment 1's results, we analysed by fitting a normal distribution function using the following formula.

$$p(\text{simultaneous}) = \text{amplitude} \times \exp\left[-\frac{(\text{SOA} - \text{PSS})^2}{2\sigma^2}\right] \qquad \text{(S2)}$$

Figure S1 shows the participants' PSSs under visual leading vs. auditory leading conditions in Experiment 1, estimated by the above model. There was a significant difference between the PSS in the visual leading and the auditory leading conditions ($t(19) = 2.53$, $p = .020$, $d_D = 0.57$), similar to when PSSs were estimated by the four-parameter model (see the main text). This difference was significant even after excluding data considered outliers according to

the criterion presented in the previous section ($t(18) = 2.31$, $p = .033$, $d_D = 0.53$). Therefore, we concluded that changes in the fitting methods for calculating the PSSs would not affect the overall conclusions of Experiment 1.

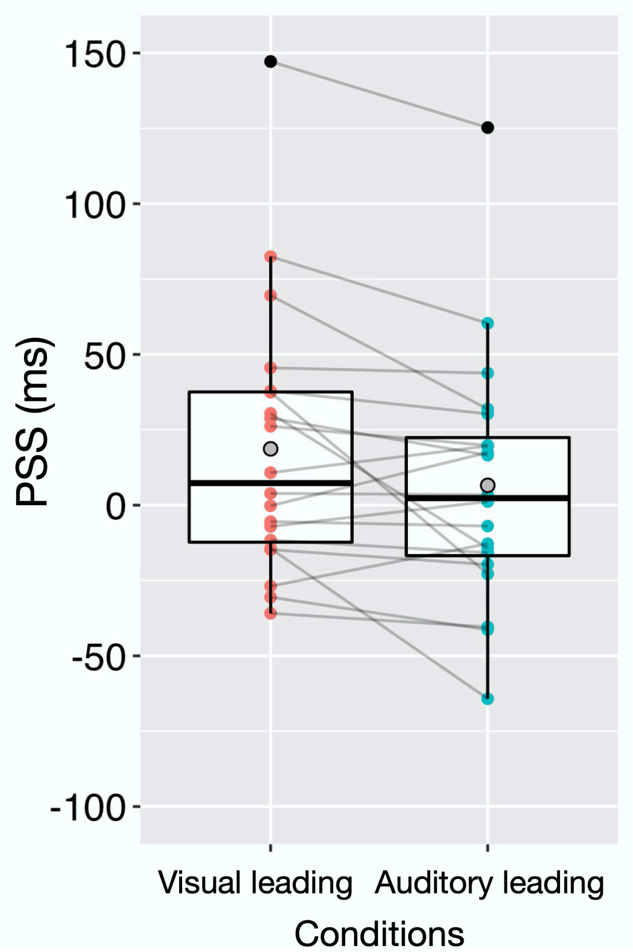

Figure S1. PSS data as a function of adaptation condition (visual leading or auditory leading) in Experiment 1, estimated by fitting a normal distribution function. Boxes represent the interquartile ranges (IQRs), central horizontal lines the medians, and grey circles the means. Magenta and cyan points represent the PSS data of individual participants. The vertical lines represent the ranges from “the first quartile – 1.5 × IQR” to “the third quartile + 1.5 × IQR,” and black points indicate the PSS data outside these ranges (i.e., outliers).

Effects of outliers on the results of Experiment 2

We investigated whether the results of Experiment 2 changed if we excluded outliers from the analysis. Three participants' data were considered outliers in at least one of the four conditions based on the identical criterion as Experiment 1, which we excluded from the subsequent analysis (see Figure 4 of the main text).

The PSS values of 28 participants were then subjected to a repeated-measures ANOVA, with test stimulus condition (congruent and incongruent) and adaptation condition (visual leading and auditory leading) as within-subject variables. Neither the main effect of test stimulus condition ($F(1, 27) = 1.59$, $p = .218$, $\eta_p^2 = .056$), nor the main effect of adaptation condition were significant ($F(1, 27) = 3.06$, $p = .092$, $\eta_p^2 = .102$). However, the interaction between these conditions was significant ($F(1, 27) = 4.35$, $p = .047$, $\eta_p^2 = .139$). A post-hoc simple effect test on the interaction revealed a significant simple main effect of the adaptation condition in the congruent test stimulus condition ($F(1, 27) = 7.57$, $p = .011$, $\eta_p^2 = .219$). In contrast, the simple main effect of the adaptation condition in the incongruent test stimulus condition was not significant ($F(1, 27) = 0.36$, $p = .557$, $\eta_p^2 = .013$). Moreover, the simple main effect of the test stimulus condition was not significant in the visual leading condition ($F(1, 27) < 0.01$, $p = .970$, $\eta_p^2 < .001$). This pattern of results was identical to when the outliers were not excluded. However, unlike when the outliers were not excluded, the simple main effect of the test stimulus condition in the auditory leading condition was not significant ($F(1, 27) = 3.91$, $p = .058$, $\eta_p^2 = .127$).

The above analysis confirmed that the pattern of results in Experiment 2 was nearly identical regardless of excluding outliers or not. However, the simple main effect of congruency in the auditory leading condition, which was significant when we did not exclude the outliers,

was not significant when we excluded the outliers. However, this did not affect our interpretation of the results because we did not focus on the main effect of the test stimuli's congruency.

Effect of changing the fitting method on the results of Experiment 2.

We analysed the results by fitting a normal distribution function (equation S2) to examine the effect of different fitting methods on the results of Experiment 2. Participants' PSSs under the conditions of Experiment 2 that were estimated by the above model are shown in Figure S2. Repeated-measures ANOVA revealed that neither the main effect of test stimulus condition ($F(1, 31) = 0.36$, $p = .554$, $\eta_p^2 = .011$), nor the main effect of adaptation condition were significant ($F(1, 31) = 1.12$, $p = .300$, $\eta_p^2 = .035$). However, the interaction between these conditions was significant ($F(1, 31) = 7.85$, $p = .009$, $\eta_p^2 = .202$). A post-hoc simple effect tests on the interaction revealed a significant simple main effect of the adaptation condition in the congruent test stimulus condition ($F(1, 31) = 5.03$, $p = .032$, $\eta_p^2 = .140$). On the other hand, the simple main effect of adaptation condition in the incongruent test stimulus condition was not significant ($F(1, 31) = 0.22$, $p = .644$, $\eta_p^2 = .007$). Moreover, the simple main effect of the test stimulus condition was significant in auditory leading condition ($F(1, 31) = 4.37$, $p = .045$, $\eta_p^2 = .124$) but not in the visual leading condition ($F(1, 31) = 1.26$, $p = .270$, $\eta_p^2 = .039$). The above results confirmed that the pattern of results in Experiment 2 was identical regardless of the fitting method (four-parameter model described in the main text vs. the normal distribution function).

We also examined whether the results were consistent when outliers ($N = 3$, see Figure S2) were excluded. Neither the main effect of test stimulus condition ($F(1, 28) = 0.54$, $p = .468$, $\eta_p^2 = .019$), nor the main effect of adaptation condition were significant ($F(1, 28) = 2.08$, $p = .161$, $\eta_p^2 = .069$), but the interaction between these conditions was significant ($F(1, 28) = 7.09$,

p = .013, η_p^2 = .202). A post-hoc simple effect test on the interaction revealed a significant simple main effect of the adaptation condition in the congruent test stimulus condition ($F(1, 28)$ = 6.92, p = .014, η_p^2 = .198). In contrast, the simple main effect of the adaptation condition in the incongruent test stimulus condition was not significant ($F(1, 28) < 0.01$, p = .947, $\eta_p^2 < .001$). Moreover, the simple main effect of the test stimulus condition was neither significant in the visual leading condition ($F(1, 28)$ = 0.77, p = .388, η_p^2 = .027) nor in the auditory leading condition ($F(1, 28)$ = 4.14, p = .051, η_p^2 = .129). This pattern of results is identical to when the four-parameter model was fitted to the data and outliers were excluded. In summary, changes in the fitting methods for calculating the PSSs did not affect the overall conclusions of Experiment 2.

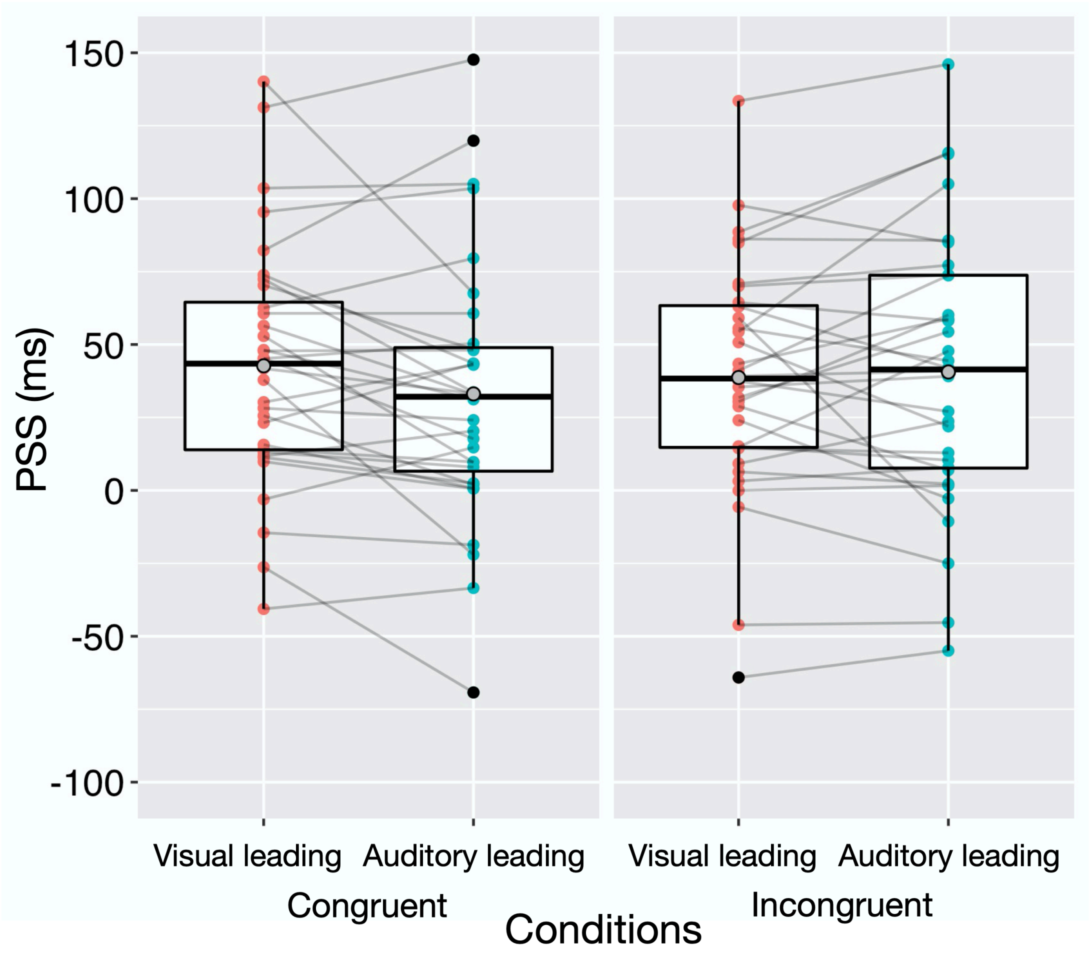

Figure S2. PSS data as a function of test stimulus condition (congruent or incongruent) and adaptation condition (visual leading or auditory leading) in Experiment 2, which we estimated by fitting a normal distribution function.
